# Paddy pest image segmentation based on multiscale attention fusion VM-UNet

**DOI:** 10.3389/fpls.2025.1700556

**Published:** 2026-01-15

**Authors:** Yunlong Zhang, Yu Shao, Ting Zhang

**Affiliations:** 1 Henan Agricultural Information Data Intelligent Engineering Research Center, SIAS University, Zhengzhou, China; 2 College of Computer, Xijing University, Xi’an, China

**Keywords:** paddy pest image segmentation (PPIS), VM-UNet, improved attention fusion (IAF), multiscale VSS (MSVSS), multiscale attention fusion VM-UNet (MSAF-VMUNet)

## Abstract

Precise paddy pest image segmentation (PPIS) in the real-time natural environments is an important and challenging research. Convolutional Neural Networks (CNNs) and Transformers are the most popular architectures for image segmentation, but they usually have limitations in modeling global dependencies and quadratic computational complexity, respectively. A multiscale attention fusion VM-UNet (MSAF-VMUNet) for PPIS is constructed. It integrates the long-range dependencies modeling ability of Visual State Space Model (VSS) and the precise positioning capability of U-Net with low computational complexity. In the model, multiscale VSS (MSVSS) block is used to capture the long-range contextual information, and improved attention fusion (IAF) module is designed for multi-level feature learning between Encoder and Decoder. Attention VSS module is introduced in the bottleneck layer to enable the model to adaptively emphasize key features and suppress redundant information. Compared with VM-UNet, MSAF-VMUNet can effectively model global-local and context relationships at the scale layer, and improve the detection performance of various pests in size and shape without increasing computational complexity. The experimental results on the paddy pest subset of the public IP102 dataset validate that MSAF-VMUNet can effectively address the key challenges in field PPIS, including small pest detection, occlusion and noise handling, and preprocessing requirements, and the PPIS presion is 79.17%, which are 15.51% and 3.39% higher than those of the traditional U-Net and the recent VM-UNet, respectively. It provides an effective and reliable solution for pest control detection system in smart agriculture.

## 1 Introduction

It is well known that paddy pests in the field seriously affect the yield and quality of rice (Abdullah et al., 2023), timely and accurate monitoring of paddy pests in the field is a prerequisite for effective prevention and control of crop diseases and pests, and paddy pest image segmentation (PPIS) in the field is crucial for dynamically monitoring pests, determining pest types, assessing the density of pests, guiding further pest control and promoting crop health (Zhang et al., 2024). However, it is confronted with numerous challenges, such as irregular and changeable pest sizes and shapes in field, low color contrast between pests and the background, blurred pest boundaries, and interference in the complex natural environments (Elmagzoub et al., 2024). In PPIS, convolution neural networks (CNNs), especially U-Net, has achieved remarkable progress due to their powerful local feature learning ability. However, U-Net and its variants usually have limitations in modeling global dependencies due to the intrinsic local convolution operation (Wu et al., 2021). Transformer and vision Transformer (ViT) have emerged as a prominent architecture for capturing long-range dependencies due to the global self-attention mechanism (Lu et al., 2025b; Lu et al., 2025a). Recent studies on Convolution-Transformer architectures (Yin et al., 2024) highlight the trend of hybrid models, which motivates our integration of multiscale convolutional operations with state space models. To overcome the limited receptive fields of CNNs and the high computational complexity of ViTs, Vision Mamba U-Net (VM-UNet) has emerged as a promising approach in complex image segmentation tasks (Wang et al., 2024). It combines a hybrid architecture of U-Net and State Space Model (SSM). It can capture long-range dependencies with linear computational complexity, but it does not specifically handle multiscale feature learning, which leads to flaws when analyzing various pests with different sizes and shapes (Rahman et al., 2024). Inspired by VM-UNet, a multiscale attention fusion VM-UNet (MSAF-VMUNet) for PPIS is constructed. To improve the results of PPIS, a multiscale attention fusion VM-UNet (MSAF-VMUNet) for PPIS is constructed. It follows the encoder-decoder network structure to capture both local features and long-range contexts in an efficient way. Unlike VM-UNet, which relies on standard VSS blocks, MSAF-VMUNet introduces the Multiscale VSS (MSVSS) block, which uniquely integrates Multi-Scale Dilated Convolutions (MSDC) with SS2D operation. It is a synergistic combination of the Improved Attention Fusion (IAF) module and the Attention VSS (AVSS). IAF dynamically filters encoder features before fusion, while the AVSS acts as a global feature refiner in the bottleneck.

The main contributions of this paper are listed as follows,

- MSVSS and IAF are used to integrate and enhance feature representations of Encoder and Decoder through multiscale channel-spatial attention mechanism.
- AVSS is introduced in the bottleneck layer to enable the model to adaptively emphasize important features and suppress redundant information based on the input image content, enhancing the discriminative ability in complex scenarios.
- MSAF-VMUNet is verified on the paddy pest subset of IP102 dataset, and the results validate that it is effective for PPIS.

The remainder of this paper is organized as follows. Section 2 briefly introduces the related work. Section 3 describes the proposed MSAF-VMUNet in detail. Section 4 presents the experimental results and corresponding discussions. Finally, Section 5 summarizes the paper and points out the future work.

## 2 Related work

Crop pest image segmentation (CPIS) involves pixel-level classification to identify detailed structures and information in pest images. With the rapid development of computer vision and deep learning (DL) technologies, traditional CPIS methods that rely on handcraft features have been replaced by deep learning-based methods, and many CPIS methods have been continuously presented, which are roughly classified into three categories: Machine learning(ML) and deep learning(DL), Transformers and Mamba-based methods (Abdullah et al., 2023).

### 2.1 ML and DL-based methods

Over the past decade, many ML and DL models have been widely applied to various computer vision fields, including anomaly detection (Tu et al., 2025), and rice row detection in paddy fields (Chen et al., 2025). Chithambarathanu et al (Chithambarathanu and Jeyakumar, 2023). reviewed the utilization of ML techniques such as Random Forest (RF), Support Vector Machine (SVM), Decision Tree (DT), and Naive Bayes (NB) in recent years, and introduced in detail the research progress of DL models in the field of crop pest and disease identification, including CNN, long Short-Term memory (LSTM), Deep CNN (DCNN), and deep belief Network (DBN). To identify and classify pests of citrus plants, rice and cotton, as well as many detection methods. Wang et al (Wang et al., 2023). proposed a three-scale CNN with attention for crop pest and disease recognition, aiming to address the issue of insufficient accuracy in existing methods. Guo et al (Guo et al., 2024). comprehensively reviewed the pest detection and identification methods based on CNN, outlined the main CNN architectures and standard evaluation metrics in this field, investigated a series of detection and identification algorithms developed in recent years, including detailed method explanations and corresponding performance results, and discussed the main challenges currently faced by deep learning-driven pest detection systems and the future research directions. U-Net is one of the most remarkable CNN models, known for its symmetrical encoder-decoder structure and skip connection, and has been widely applied to crop disease and pest detection tasks. Biradar et al (Biradar and Hosalli, 2024). proposed a hybrid U-Net framework for accurate CPIS and detection. The framework integrates image preprocessing with a DenseNet-77-enhanced U-Net for high-precision CPIS, reducing computational complexity versus traditional U-Net. To improve the detection accuracy of field insect pests, Wang et al (Wang et al., 2024). constructed a dilated multiscale attention U-Net by using the advantages of ResNet, dilated convolution and Inception module. Zhang et al (Zhang et al., 2025). proposed a multiscale fusion network for robust pest detection, in which the image-text fusion module is used for joint modeling of vision and text features, and the image-text converter is used to reconstruct fine-grained details across multiscale to handle challenging backgrounds.

The above CNN-based methods have achieved remarkable performance in the field of CPIS and pest detection, but due to the fact that pest targets are usually small in size and have few features, as the depth of the CNN network increases, the key feature information of pests will gradually decay, seriously restricting the model ability to distinguish dense small pest targets.

### 2.2 Transformer-based methods

Transformer and ViT have been widely applied to complex image segmentation tasks, and have shown significant potential in CPIS. Their main part is self-attention mechanism, where the used *Q, K*, and *V* matrices are obtained by the linear transformation of the output. Fu et al (Fu et al., 2024). proposed a CPIS method based on the improved ViT model. In the model, self-attention mechanism is used to effectively select the region with the most obvious characteristics. Saranya et al (Lu et al., 2025b). presented an efficient agricultural pest classification method using ViT with hybrid pooling multihead attention. It outperforms both CNN and ViT models. Liu et al (Liu et al., 2025). proposed an end-to-end pest detection method, which utilizes feature representation compensation and regional pale shape self-attention to compensate for the loss of feature information caused during downsampling. To leverage the advantages of both Transformer and U-Net architectures, Lu et al (Lu et al., 2025). constructed a Cross-Attention TransU-Net (CATransU-Net) model is for paddy pest detection by combining U-Net and Transformer. The graph frequency learning approaches in anomaly detection (Gan et al., 2022) further demonstrate Transformer capability in handling complex spatial relationships, which is particularly relevant for pest segmentation in cluttered backgrounds.

Compared with CNN and U-Net models, Transformers and ViT demonstrate superior capability in modeling long-range dependencies and capturing global contextual information for crop pest detection (Fang et al., 2024). However, their quadratic computational complexity of *O*(*n*²) severely limits practical deployment in agricultural scenarios that require real-time and high-resolution image analysis.

### 2.3 Mamba-based methods

Recent advances in agricultural computing highlight the effectiveness of hybrid architectures and state-space models, such as lightweight CNN-Transformer fusions for plant disease identification and VSS models like VM-UNet for efficient long-range context capture. These approaches demonstrate the value of combining local feature extraction with global dependency modeling while maintaining computational efficiency. Mamba is a novel DL model based on SSM. It is validated to be more effective with lower parameters than Transformers, and has been successfully applied to computer vision tasks. U-Mamba is a hybrid local-global mechanism. It uses convolutional layers for local feature extraction and then uses Mamba blocks for global modeling (Ma et al., 2024). Vision Mamba (VMamba) inherits the advantages of CNNs and ViTs through VSS, can capture global context information by introducing Mamba into the encoder, while retaining the skip-connection and symmetrical codec structure of U-Net to maintain the ability of local feature integration (Liu et al., 2024). Recent applications in agricultural vision, such as two-pathway instance segmentation for rice rows (Qureshi et al., 2024) and efficient underwater target detection (Wang et al., 2025), demonstrate the potential of advanced architectures in agricultural computer vision tasks. VM-UNet, as an alternative architecture for PPIS, inherits the advantages of U-Net and VMamba (Wang et al., 2024). It is a novel architecture, and has shown great potential in the field of image segmentation due to modeling long-range dependencies with linear computational complexity (Cao et al., 2025; Zhao et al., 2025). InsectMamba is a novel pest classification approach that integrates SSM, CNN, multi-head self-attention mechanism (MSAM), and multi-layer perceptron (MLP) into a hybrid SSM block (Wang et al., 2024). This integration promotes the comprehensive vision feature extraction by taking advantage of the strengths of each coding strategy. The model is evaluated against strong competitors on five pest classification datasets.

While CNN and U-Net models struggle to capture long-range dependencies—essential for detecting pests in complex backgrounds and fine-grained details—Vision Transformers (ViTs) excel at modeling global context, yet their quadratic computational complexity O(n²) hinders real-time deployment in agricultural applications. Existing PPIS methods struggle to simultaneously resolve three key issues: preserving fine-grained features of small pest targets, distinguishing densely distributed pests, and suppressing interference from complex backgrounds. To address these challenges, we propose a Multiscale Attention Fusion VM-UNet (MSAF-VMUNet). The model leverages integrated multiscale feature fusion and attention mechanisms to enhance representational capacity and contextual understanding. It introduces a Multiscale VSS (MSVSS) block and an Improved Attention Fusion (IAF) module to tackle multiscale pest variations and complex background noise in field environments. The proposed model is extensively evaluated on a paddy pest image subset from the public IP102 benchmark, demonstrating its effectiveness in real-world agricultural scenarios (Kang et al., 2025).

## 3 MSAF-VMUNet

**Figure 1:**
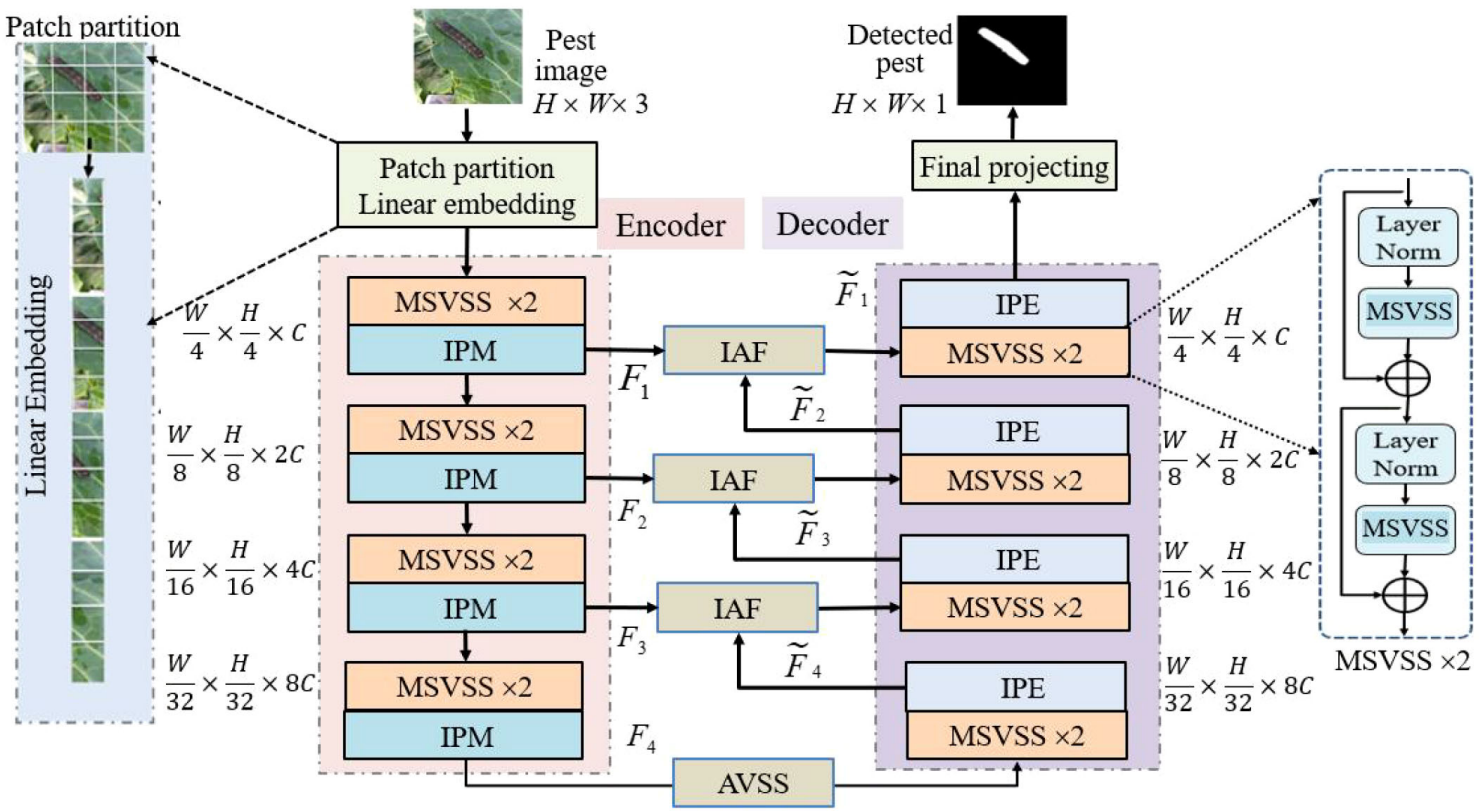
The architecture of MSAF-VMUNet.

The overall architecture of the proposed MSAF-VMUNet is shown in **Figure 1**, including three main modules specifically to address the challenges in paddy pest image segmentation: the Multiscale VSS (MSVSS) block (in Encoder/Decoder) as the fundamental feature extractor, the Improved Attention Fusion (IAF) module (in Skip Connections) as a dynamic feature filter, and the Attention VSS (AVSS) module (in Bottleneck) as a global feature refiner at the most abstract level.

**Figure 2:**
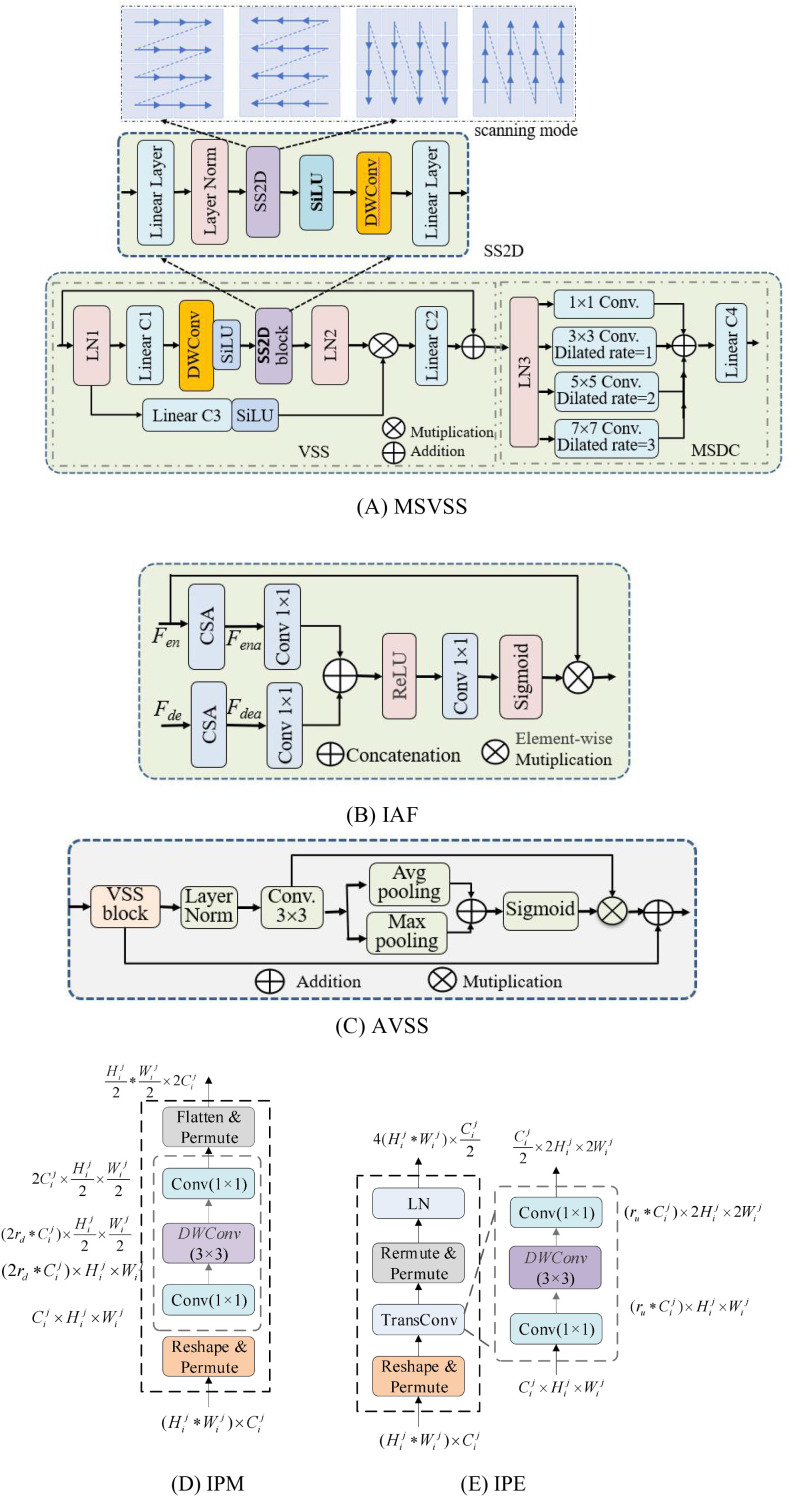
Main components of MSAF-VMUNet: **(A)** MSVSS module; **(B)** IAF module; **(C)** AVSS module; **(D)** IPM module; **(E)** IPE module.

MSVSS, IAF and AVSS, along with the Improved Patch Merging (IPM) and Improved Patch Expanding (IPE) operations, form a coordinated MSAF-VMUNet. Their structures are shown in **Figure 2**. (1) MSVSS uses dilated convolutions and the selective scan mechanism to capture rich, multi-scale contextual information, thereby effectively handling pests of diverse sizes and shapes; (2) IAF replace standard skip connections to dynamically filter and accentuate semantically critical features from the encoder prior to their fusion with the decoder, which suppresses background interference and irrelevant details; and (3) AVSS positioned in the bottleneck acts as a global feature refiner, adaptively emphasizing crucial patterns while compressing redundant information. This synergistic integration of components establishes a cohesive framework that progressively extracts, refines, and reconstructs features, achieving precise pixel-wise segmentation of paddy pests in complex field environments while maintaining linear computational complexity.

In the patch partition and embedding layer, the input image $Im\in R^{H\times W\times 3}$ is divided into non-overlapping patches of size 4×4, and each patch is linearly projected into a *C*-channel space, with default *C* = 96, yielding an embedded representation $F\in R^{H/4\times W/4\times C}$. Encoder consists of four dual-MSVSS modules, each following a IPM operation applied to reduce spatial dimensions (height and width) while increasing channel depth. Decoder is composed of four dual-MSVSS modules, each following an IPE operation to upsample the feature maps, decreasing channel count and increasing spatial resolution. AVSS aims to capture global-local features and compress redundant features. Skip connections are implemented through IAF module. Final Projection layer performs 4× upsampling via patch expanding to restore the original input resolution, followed by a linear projection to match the number of output channels for segmentation.

### 3.1 MSVSS

MSVSS in the encoder and decoder is the core module of MSAF-VMUNet, as depicted in **Figure 2A**, consisting of VSS and multiscale dilated convolution (MSDC). Its processes include layer normalization (*LN*), linear projection (*Lin*) depth-wise separable convolution (*DWConv*), nonlinear activation (*SiLU*), 2D-Selective-Scan (*SS2D*), and *MSDC*. The process of MSVSS is as show in Equation 1:

(1) $$\begin{array}{l} F_{out}^{MSVSS}=Lin4(MSDC(LN3(VSS(V_{in}))))\\ VSS(V_{in})=Lin2(LN2(SS2D(SiLU(DWConv((Lin1(LN1(V_{in}))))))⊗V_{ls})+V_{in}\\ V_{ls}=SiLU(Lin3(LN1(V_{in})))\\ MSDC(x)=(Conv_{1\times 1}(x)⊕Conv_{3\times 3}(x)⊕Conv_{5\times 5}(x)⊕Conv_{7\times 7}(x)) \end{array}$$

where $V_{in},F_{out}^{MSVSS}$ are the input and output feature maps of MSVSS, *LN*(·), *Lin*(·), *SiLU*(·), *SSM*(·) and *DWConv*(·) are layer normalization, linear projecting, SiLU activation, SS2D and DWConv operations, respectively, VSS(.) is the VSS operation.

In MSVSS, SS2D block enables comprehensive spatial feature analysis while maintaining computational efficiency through selective information processing. It consists of three components: a scan expanding operation, an S6 block, and a scan merging operation, where the scan expanding operation unfolds the input image along four different directions into sequences, i.e., top-left→bottom-right, bottom-right→top-left, top-right→bottom-left, bottom-left→top-right, and are processed by the S6 block for feature extraction, ensuring that information from various directions is thoroughly scanned, thus capturing diverse features.

### 3.2 IAF

In the traditional U-Net, Skip-Connection simply concatenates the multiscale feature map of the encoder to the upsampled feature map corresponding to the decoder. This approach may generate redundant information, noise, and background information irrelevant to the segmentation task. MSAF-VMUNet introduces an IAF module into Skip-Connection to achieve the attention feature fusion between the encoder and the decoder, enhancing the modeling ability and realizing more accurate multiscale feature fusion. Its structure is shown in **Figure 2B**. Its process is as show in Equation 2:

(2) $$\begin{array}{l} F_{IAF}=Sig(Conv_{1\times 1}(\text{ReLU}(F_{en}^{'}⊕F_{de}^{'})))⊗F_{en}\\ F_{en}^{'}=Conv_{1\times 1}(CSA(F_{en})),F_{de}^{'}=Conv_{1\times 1}(CSA(F_{de}))\; \end{array}$$

where $F_{en}$ and $F_{de}$ are the features of the encoder and the decoder, $F_{IAF}$ is the output of IAF, Sig(.)is Sigmoid function, $Conv_{1\times 1}$ is 1×1 convolutional operation, CSA(.) is channel-spatial attention operation, $Conv_{1\times 1}$ is employed to project feature maps into the same feature space.

The core of the IAF module is the custom Channel-Spatial Attention (CSA) operation, which is sequentially applied to first refine channel-wise feature responses and then highlight spatially informative regions. As shown in **Figure 2**, given an input feature map, the CSA operation proceeds through two distinct steps:

Channel Attention Path: The global average pooling and global max pooling in parallel are used to generate two distinct spatial context descriptors. These descriptors are then fed into a shared multi-layer perceptron (MLP) with a single hidden layer. The outputs of the MLP are merged by element-wise summation and passed through a sigmoid activation function to generate a channel attention vector. This vector is then multiplied with the original input feature map to produce channel-refined features.

Spatial Attention Path: The channel-refined features are then passed to the spatial attention component. We apply both average-pooling and max-pooling along the channel dimension to generate two 2D spatial feature maps. These maps are concatenated and processed by a standard 7×7 convolutional layer, followed by a sigmoid function, to generate a spatial attention map. Finally, this map is multiplied with the channel-refined features to yield the final output of the CSA module, which dynamically emphasizes informative features across both dimensions.

This design differs from Convolutional Block Attention Module (CBAM) in its use of a larger 7×7 convolution kernel in the spatial path, which we found provides a broader contextual field for more effectively integrating global information, a crucial factor for accurately locating pests of varying sizes in complex field backgrounds.

### 3.3 AVSS

In U-Net, the bottleneck layer serves as a bridge between the encoder and the decoder. VM-UNet typically only uses conventional VSS modules for sequence modeling at the bottleneck layer. Although VSS effectively captures long-range dependencies through SS2D, its feature processing remains relatively uniform and static. It lacks a mechanism to dynamically emphasize important features and suppress secondary or interfering features. AVSS is introduced in the bottleneck layer of VM-UNet, aiming to enable the model to adaptively emphasize key features and suppress redundant information based on the input image content, enhancing the discriminative ability in complex scenarios. Its structure is shown in **Figure 2C**. Its process is as show in Equation 3:

(3) $$\begin{array}{l} F_{out}^{AVss}=Sig(Avgp(F^{'})⊕Maxp(F^{'}))⊗F^{'}⊕VSS(F_{in}^{AVss})\\ F^{'}=Conv_{3\times 3}(LN(VSS(F_{in}^{AVss}))) \end{array}$$

where $F_{in}^{AVss}$ and $F_{out}^{AVss}$ are the input and output feature maps of AVSS, *Avgp*(·) and *Maxp*(·) avg-pooling and max-pooling, $Conv_{3\times 3}$ is 3×3 convolutional operation.

### 3.4 LPM and LPE

LPM in the encoder captures long-range dependencies while gradually reducing the spatial dimension, effectively compressing the input into multiscale representations. LPE in the decoder maintains the ability to learn complex spatial relationships and enhances the adaptability to PPIS of various pests. Their structures are shown in **Figures 2D, E**, and their operation processes are as show in Equations 4, 5:

(4) $$F_{out}^{IPM}=f_{flatten}(f_{conv1\times 1}(f_{DSconv3\times 3}(f_{conv1\times 1}(f_{reshape}(x_{in})))))$$

(5) $$F_{out}^{IPE}=LN(f_{reshape}(f_{conv1\times 1}(f_{Transconv3\times 3}(f_{conv1x\times 1}(f_{reshape}(y_{in}))))))$$

where $x_{in}$ and $F_{out}^{IPM}$are the input and output of IPM, $y_{in}$and $F_{out}^{IPE}$ are the input and output of LPE, $f_{flatten}$, $f_{DSconv3\times 3}$, $f_{reshape}$, $f_{conv}$and $f_{Transconv3\times 3}$ are the feature map flattening, *DWConv*(3×3), feature reset, convolution and transposed convolution operations, respectively.

### 3.5 Loss function

Following the widely used metrics to train DLmodel, a hybrid loss function Loss combining multiclass cross-entropy loss $\;L_{MSCE}$ and Dice Similarity Coefficient (DSC) loss $L_{Dice}$ is used to train the model and optimize its hyperparameters, where $\;L_{MSCE}$ is used to ensure pixel-wise classification accuracy across cardiac structures, addressing class imbalances, and $L_{Dice}$ is used to maximize the overlap between predicted and ground-truth regions. They are defined as show in Equations 6–8:

(6) $$Loss=\sigma L_{MSCE}+(1-\sigma )L_{Dice}$$

(7) $$L_{MSCE}=-\frac{1}{N}\sum_{i=1}^{N}\sum_{c=1}^{C}y_{i,c}log_{e}(p_{i,c})$$

(8) $$L_{Dice}=1-\frac{2\lceil X\cap Y\rceil }{\left\lceil X\rceil +\lceil Y\right\rceil }$$

where *N* and *C* are the total number of samples and the total number of categories, respectively, σ is an adjustable parameter with default 0.6, $y_{i,c}$ is an indicator function that takes 1 if sample *i* belongs to class *c*, and 0 otherwise, $p_{i,c}$ is the predicted probability of the model that sample *i* belongs to class *c*, X and Y are the ground truth labels and predicted results, respectively, X∩Y is the intersection between *X* and *Y*.

### 3.6 Computational complexity

Considering that the number of pixels *n* is much greater than parameters such as the number of images and the number of feature channels. MSAF-VMUNet consists of an embedding layer, MSVSS, AVSS, IAF and Linear projection. Their computational complexities are all *O*(*n*). Therefore, the complexity of MSAF-VMUNet is linear *O*(*n*) relative to the number of pixels *n*. This is more efficient than traditional Transformers with quadratic complexity *O*(*n*²), making MSAF-VMUNet highly scalable PPIS.

To highlight the differences between MSAF-VMUNet and key baseline models (U-Net, VM-UNet, and TransUNet), **Table 1** presents a comparison of the backbone design, skip connection mechanism, attention mechanism, and multi-scale processing in these models.

**Table 1:** Architectural comparison between MSAF-VMUNet and other segmentation models.

Model	Backbone	Skip connection	Attention mechanism	Multiscale handling	Computational complexity
U-Net	CNN	Concatenation	N/A	encoder depth	O(n)
TransUNet	ViT+CNN	Concatenation	Self-Attention	Patches & CNN layers	O(n²)
VM-UNet	VSS	Concatenation or Addition	N/A (inherent in VSS)	encoder depth	O(n)
Ours	**MSVSS**	**IAF Module**	**AVSS + CSA**	**MSVSS + IAF**	**O(n)**

The bold values provided is about model of MSVSS proposed in the paper.

From **Table 1**, it is found that the improved MSAF-VMUNet is very different from three key baseline models U-Net, VM-UNet and TransUNet.

## 4 Experiment results and analysis

In this section, a large number of comprehensive experiments are conducted on the paddy pest image set in dataset IP102 using MSAF-VMUNet. To verify its effectiveness in PPIS task, it is compared with U-Net, VM-UNet (Zhao et al., 2025), and 5 state-of-the-art PPIS models, including U-Net with hybrid deep learning mechanism (HU-Net) (Biradar and Hosalli, 2024), dilated multiscale attention U-Net (DMSAU-Net) (Wang et al., 2024), YOLOv5 detector combined with a segmentation technique (SYOLOv5) (Qureshi et al., 2024), YOLO-DBS (Wang et al., 2025), Transformer-based model with feature compensation and local information enhancement (Trans-FCLIE) (Liu et al., 2025), CATransUNet (Lu et al., 2025), and hybrid semantic segmentation network based on vision Mamba (CVMH-UNet) (Wang et al., 2025), where HU-Net and DMSAU-Net are two modified U-Nets, Trans-FCLIE and CATransUNet are two modified Transforms, and CVMH-UNet and MSAF-VMUNet are two modified VM-UNets.

### 4.1 Dataset

**Figure 3:**
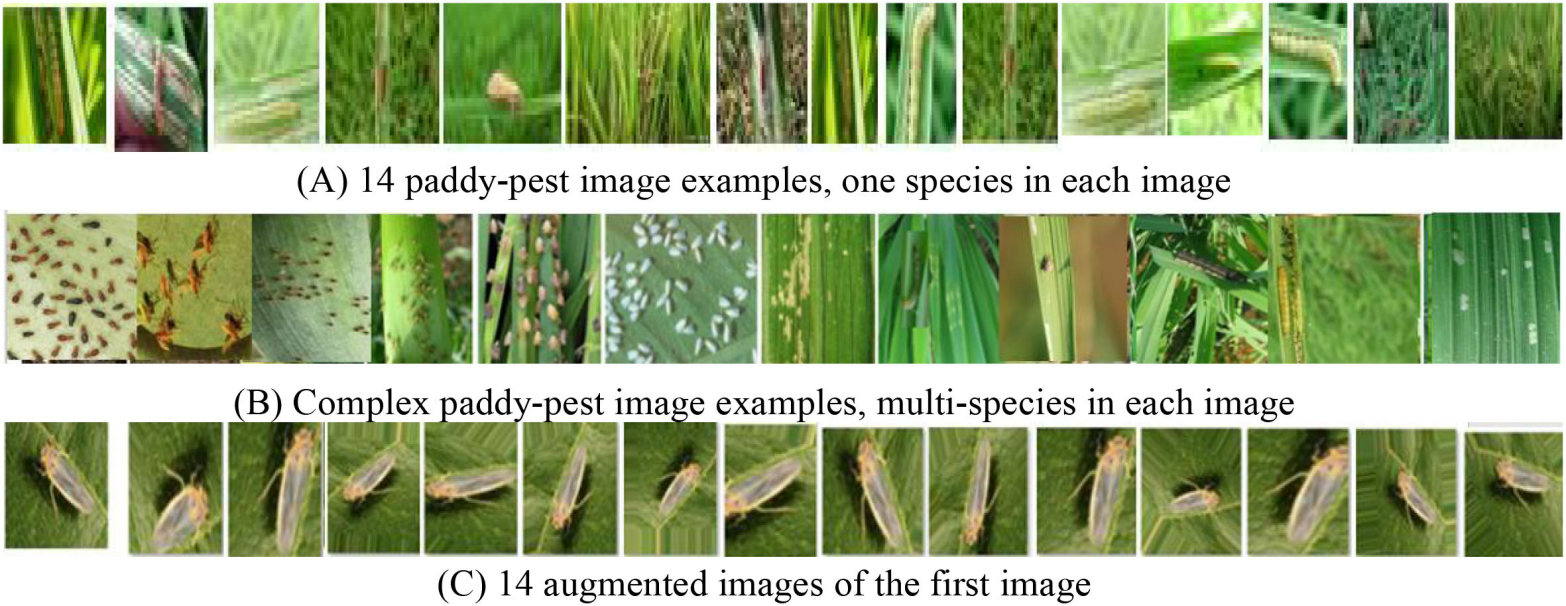
Paddy pest image samples, distribution and augmentation in IP102 dataset: **(A)** 14 single-species examples; **(B)** Complex multi-species examples; **(C)** 14 augmentations of the first image.

The IP102 dataset (https://github.com/xpwu95/IP102) is a specialized large-scale benchmark for insect pest detection, recognition and image segmentation in agriculture. It has over 75,000 images belonging to 102 categories (Wang et al., 2024). It exhibits a natural long-tail distribution, reflecting real-world data imbalance where some pest categories have significantly more samples than others. Approximately 19,000 images include bounding box annotations and Ground Truths for pest detection and PIS tasks. IP102 includes 8,415 paddy pest images from 14 classes, characterized by significant scale and sizes variations with complex backgrounds. All the images of rice pests in the IP102 dataset were collected in highly complex field conditions, characterized by: (1) The target pests show significant appearance changes at different life stages, (2) The background clutter and visual noise are relatively large, (3) Interference from overlapping elements, (4) The types of crops and plant structures are diverse, and (5) Different growth stages of crops can change the visual environment. These complexities require advanced computer vision methods that can simultaneously capture global context relationships and fine-grained local features to achieve accurate PPIS and precise detection in real-world agricultural environments. Samples display pests at different life stages (egg, larva, pupa, adult), increasing intra-class variance, as shown in **Figures 3A and B**.

The data augmentation approaches are applied to artificially balance the dataset to simulate the vast diversity of real-world field conditions:

- Random Rotation (± 30°): Accounts for varying camera orientations and pest poses, encouraging rotational invariance.
- Random Horizontal and Vertical Flip (p=0.5): Models the symmetric appearance of pests and different plant orientations.
- Random Scaling (0.8 to 1.2×): Directly addresses the critical challenge of scale variation by simulating pests at different distances from the camera.
- Brightness & Contrast Adjustment (± 20%): Simulates drastic lighting changes in the field, from bright sunlight to deep shadow.
- Color Jitter (± 10% on saturation): Compensates for variations in plant color and lighting color temperature.
- Gaussian Blur (kernel size up to 5×5): Helps the model generalize to slightly out-of-focus images, a common issue in dynamic field capture.

The augmented images are shown in **Figure 3C**. The augmented dataset has a total of 12,000 images.

In the following experiments, 5-fold cross-validation strategy on the augmented dataset. The specific protocol is as follows. The experiment is run five times. In each run, four folds (80% of the data) are used for training, and the remaining one fold (20%) are used for validation. This process is repeated until each of the five folds had served as the validation set exactly once. The performance metrics reported in the experiments are the mean and standard deviation across all five validation folds.

### 4.2 Experimental set

MSAF-VMUNet is initialized with random weights, with convolutional layers leveraging pre-trained weights from the public ImageNet dataset to enhance feature extraction capabilities. All experiments are conducted under the following uniform hardware and software configurations.

Operating System: Windows 10; Hardware: Xeon E5-2667v3 CPU, Quadro M4000 GPU; Software Stack: Python 3.8.8, PyTorch 1.10, CUDA 11.3.

The training protocol employed the following hyperparameters.

Loss Function: Cross-Entropy Loss; Optimizer: Stochastic Gradient Descent (SGD); Initial Learning Rate: 0.0001; Batch Size: 15; Momentum: 0.5; Weight Decay: 0.00001; Total Iterations: 3,000; Validation Method: 5-Fold Cross-Validation.

Due to the challenges of small pest regions and severe class imbalance in the set of paddy pest images in IP102 dataset, Precision, Recall and mean Intersection over Union (mIoU) metrices are adopted, where mIoU is one of the most core and important evaluation metrics in the field of image segmentation. They are calculated as show in Equation 9:

(9) $$Precision=\frac{T_{P}}{T_{P}+F_{P}}\times \;100\%,\;Recall=\frac{T_{P}}{T_{P}+F_{N}}\times 100\%,\;mIOU=\frac{T_{P}}{T_{P}+F_{P}+F_{N}}\times 100\%$$

where *TP*, *FP* and *FN* denote true positives, false positives, and false negatives, respectively.

### 4.3 Implementation details for baselines

Nine baseline models (U-Net, HU-Net, DMSAU-Net, SYOLOv5, YOLO-DBS, Trans-FCLIE, CATransUNet, VM-UNet, and CVMH-UNet) are simply introduced as follows.

U-Net is a baseline encoder-decoder architecture widely used in image segmentation tasks.

HU-Net is hybrid U-Net by combining DenseNet-77 UNet, CNN and a Gated Recurrent Unit (GRU).

In DMSAU-Net, dilated Inception instead of the convolution layer in U-Net is used to extract the multi-scale features of insect pest images, and an attention module is added to its decoder to focus on the edge of the insect pest image.

SYOLOv5 is a detector combined with a segmentation technique.

YOLO-DBS is an Efficient Target Detection in Complex Underwater Scene Images based on Improved YOLOv8.

Trans-FCLIE is an end-to-end Transform based pest detection method using feature representation compensation (FRC) and regional pale-shaped self-attention (RPSA) to enhance the local information.

CATransUNet is a Cross-Attention TransU-Net, consisting of encoder, decoder, dual Transformer-attention module (DTA) and cross-attention skip-connection (CASC).

VM-UNet is a vision Mamba-based U-Net variant designed for medical image segmentation but adapted here for PPIS.

CVMH-UNet is an image segmentation method through VMamba and Multiscale Multi-Frequency Feature Fusion to fully capture global information from multiple directions, and by incorporating CNN branches to overcome the constraints of VMamba in acquiring local information.

They are trained and evaluated under the exact same experimental setup as our MSAF-VMUNet, including,

Ø Identical data augmentation strategies.
Ø The same input image resolution.
Ø The same optimizer (SGD), initial learning rate (0.0001), batch size (15), and total number of iterations (3,000).
Ø The same hybrid loss function (Equation 6).
Ø The same 5-fold cross-validation procedure and hardware/software environment.

### 4.4 Vision pest segmentation results

**Figure 4:**
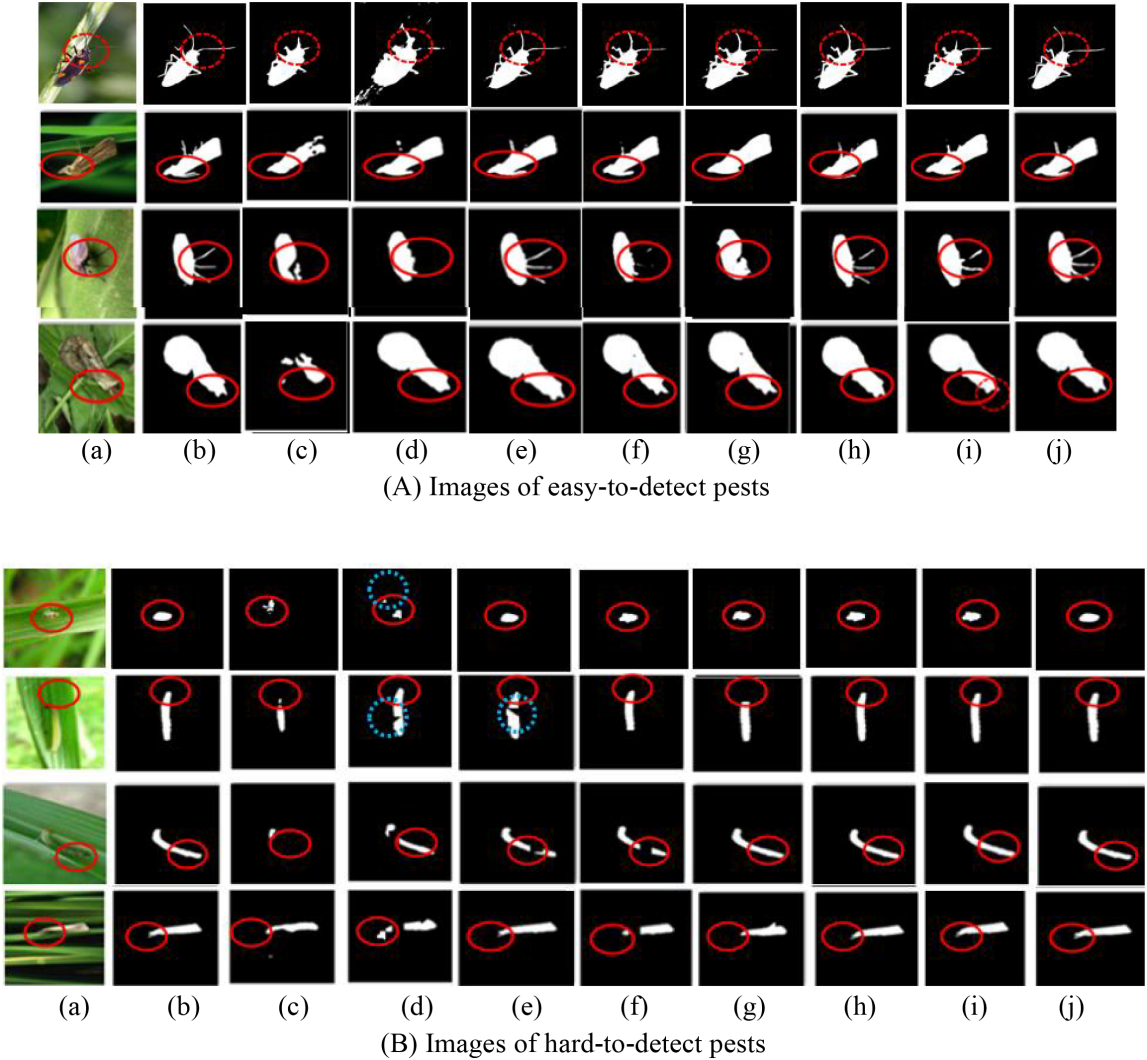
Segmented pest image samples by 8 models: **(A)** Easy-to-detect pest images; **(B)** Hard-to-detect pest images (subfigures (a)-(j)). Red circles: detailed comparison regions; blue circles: false comparison regions.

MSAF-VMUNet is compared with the state-of-the-art PPIS models. All models are trained for 3,000 iterations and evaluated on PPIS tasks, particularly focusing on rice pest images. The visual results on both simple and complex PPIS are in **Figure 4**, where (a) original images, (b) Ground truths, (c) U-Net, (d)HU-Net, (e) DMSAU-Net, (f) Trans-FCLIE, (g) CATransUNet, (h) VM-UNet, (i) CVMH-UNet and (j) MSAF-VMUNet.

As observed in **Figure 4**, MSAF-VMUNet demonstrates superior performance in accurately localizing tiny pests and effectively suppressing complex background interference, validating its strong generalization capability in real-world agricultural scenarios. For the relatively simple scenario shown in **Figure 4A**, where pest targets are large and distinct, all models except U-Net successfully detect the pests, and the most complete segmented results by MSAF-VMUNet clearly preserve fine-grained features such as pest antennae and legs. In more challenging and complex scenarios, as illustrated in **Figure 4B**, where pest targets are visually subtle and ambiguous, models like U-Net and HU-Net struggle to detect pests, whereas Trans-FCLE, CVMH-UNet, and MSAF-VMUNet maintain robust and complete detection performance.

**Figure 5:**
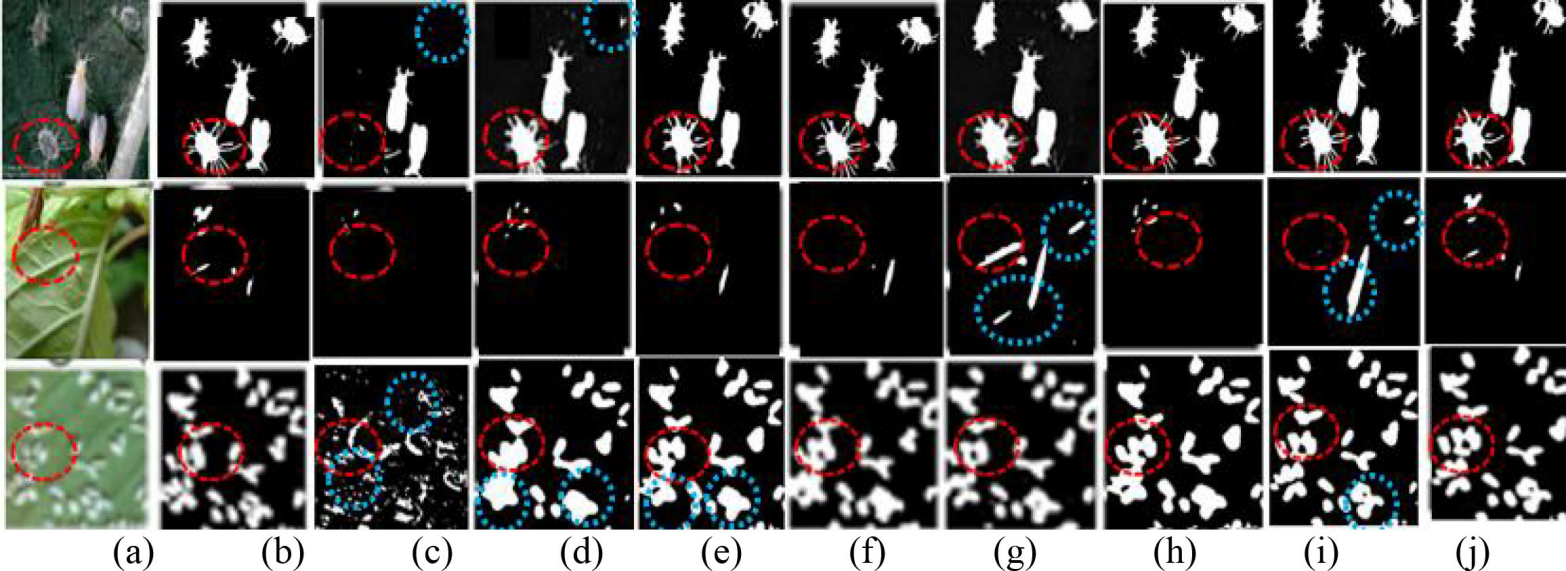
Segmented pest images: **(a)** original; **(b-j)** results of 8 models. Red circles: detailed comparison regions, blue circles: false comparison regions.

To validate the generalization of MSAF-VMUNet, PPIS experiments are conducted on challenging multi-pest images with the following characteristics, such as complex backgrounds, fuzzy boundaries, small and multiscale pests, and low foreground-background contrast. The segmented pests by 8 models are illustrated in **Figure 5**, where (a) original images, (b) Ground truths, (c) U-Net, (d) HU-Net, (e) DMSAU-Net, (f) Trans-FCLIE, (g) CATransUNet, (h) VM-UNet, (i) CVMH-UNet, and (j) MSAF-VMUNet. From **Figure 5a**, it is seen that these original images have the characteristics of complex, fuzzy, small scale, multiscale, complex background, little difference between pest and background, and there are many tiny pests in the last image.

As shown in **Figure 5j**, MSAF-VMUNet achieves the best segmented performance, with its segmented multi-pest and tiny-pests close to the ground truths. In contrast, the segmentation outputs of U-Net, HU-Net, and DMSAU-Net exhibit incompleteness, overlapping, and false pest pixels, along with missed small pests. Both Trans-FCLE and MSAF-VMUNet outperform other models. CATransUNet is prone to over-segmentation issues, likely attributable to the localized nature of convolutional operations limiting their contextual modeling capacity.

### 4.5 Quantitative results

To quantitatively validate the effectiveness of MSAF-VMUNet, it is compared with the above models, SYOLOv5 and YOLO-DBS. **Table 2** shows their Precision, DSC and Training time.

**Table 2:** The PPIS results of 10 models.

Results Methods	Precision	Recall	mIOU	Training time (h)
U-Net	64.66	60.35	52.18	11.83
HU-Net	73.31	69.85	61.45	12.46
DMSAU-Net	74.37	71.95	63.25	16.35
SYOLOv5	74.68	72.61	63.77	17.62
YOLO-DBS	74.86	73.18	64.03	17.45
Trans-FCLIE	75.09	74.10	64.89	16.74
CATransUNet	74.56	76.15	65.41	16.09
VM-UNet	75.78	73.27	64.15	**6.24**
CVMH-UNet	76.60	73.15	64.28	7.32
MSAF-VMUNet	**79.17**	73.42	66.58	7.67

The bold values provided is the precision result of the method MSAF-VMUNet proposed in the paper.

The quantitative results in **Table 2** demonstrate the superior performance of MSAF-VMUNet, which achieves the highest Precision (79.17%) and mIoU (66.58%) among all compared models. While its Recall (73.42%) remains competitive, the significant lead in precision underscores its enhanced ability to minimize false positive detections—a critical attribute for practical pest monitoring. Notably, MSAF-VMUNet maintains high efficiency, with a training time (7.67 hours) far lower than that of Transformer-based models like Trans-FCLIE and detection-oriented models like SYOLOv5, and is comparable to other Mamba-based approaches such as VM-UNet. This confirms that our model offers an optimal balance between segmentation accuracy and computational efficiency, making it well-suited for real-world agricultural applications.

### 4.6 Analysis of false negatives and recall

From **Figure 5** and **Table 2**, it is found that MSAF-VMUNet achieves superior precision and mIoU, but its recall shows a more modest improvement compared to some baselines (e.g., CATransUNet). To investigate this, we conducted a detailed analysis of false negatives (FN) to understand the model limitations in sensitivity.

We categorized the primary causes of FNs in our predictions, as illustrated in **Figure 5**. From **Figure 5**, it is seen that, (1) Extremely Small and Dense Pest Clusters: The most significant source of FNs is from pests that occupy less than 0.1% of the image area, especially when clustered together. In these cases, the model sometimes failed to separate individual pests, merging them into the background or with adjacent pests; (2) Heavy Occlusion by Debris or Plant Structures: Pests that were partially obscured by rice leaves, stems, or shadows were occasionally missed. While the MSVSS block captures long-range context, fine-grained details of heavily occluded targets remain challenging; (3) Pests with Extreme Color Similarity to Background: A small subset of pests, such as early-instar larvae, have coloration that is almost identical to the rice stem or leaf. The low color contrast led to the model incorrectly classifying these pest pixels as background. The IAF module’s role in filtering encoder features, though beneficial for precision, might be slightly over-suppressive for these ambiguous cases.

### 4.7 Stratified performance analysis

To ensure an objective and reproducible analysis, the test set is stratified using the following quantitative criteria:

1) Stratification criteria as follows.

Ø Pest Size: The test images were categorized into three groups based on the relative area of the pest pixels compared to the entire image:

Ø Small: Pest area < 1% of the image area.

Ø Medium: 1% ≤ Pest area < 5%.

Ø Large: Pest area ≥ 5%.

2) Background Complexity: To eliminate subjectivity, we employed Image Entropy as an objective metric for background complexity. It is calculated as show in Equation 10:

(10) $$Entropy=-\sum_{i=0}^{255}p(i)\log(p(i))$$

where *p*(*i*) is the probability of the *i*-th gray level in the image histogram (0-255).

Higher entropy values indicate more complex textures and information-rich backgrounds with greater randomness, while lower values correspond to smoother, more uniform backgrounds. The images are classified into two classes by Image Entropy as follows,

² Low Complexity: Image Entropy ≤ Median.

² High Complexity: Image Entropy > Median.

3) Analysis of results.

The stratified performance, measured by the DSC, is summarized in **Table 3**. For clarity and conciseness, we compare our MSAF-VMUNet against three representative baseline models: U-Net (CNN-based), Trans-FCLIE (Transformer-based), and VM-UNet (Mamba-based).

**Table 3:** Stratified analysis of segmentation performance (DCS, %).

Stratification category	U-Net	Trans-FCLIE	VM-UNet	Ours
Small (< 1%)	61.50 ± 4.80	72.15 ± 4.10	72.85 ± 3.65	78.01 ± 3.15
Medium (1-5%)	69.25 ± 3.30	80.05 ± 2.80	80.65 ± 2.55	84.12 ± 2.00
Large (≥ 5%)	75.30 ± 2.60	82.55 ± 2.10	82.20 ± 2.25	85.40 ± 1.65
Low Complexity	73.85 ± 2.35	82.25 ± 1.95	81.75 ± 2.05	85.01 ± 1.50
High Complexity	59.50 ± 5.40	71.05 ± 4.75	73.65 ± 4.10	79.28 ± 3.20

From the results in **Table 3**, it is concluded that MSAF-VMUNet is superior across all scenarios, particularly for small pests (78.01%) and complex backgrounds (79.28%). The model outperforms suboptimal models by 3.84-5.63% in these challenging categories, demonstrating the MSVSS and IAF modules’ effectiveness in accurate pest localization. These results validate MSAF-VMUNet robustness for practical agricultural applications requiring high-precision pest identification.

### 4.8 Impact of model components

**Figure 6:**
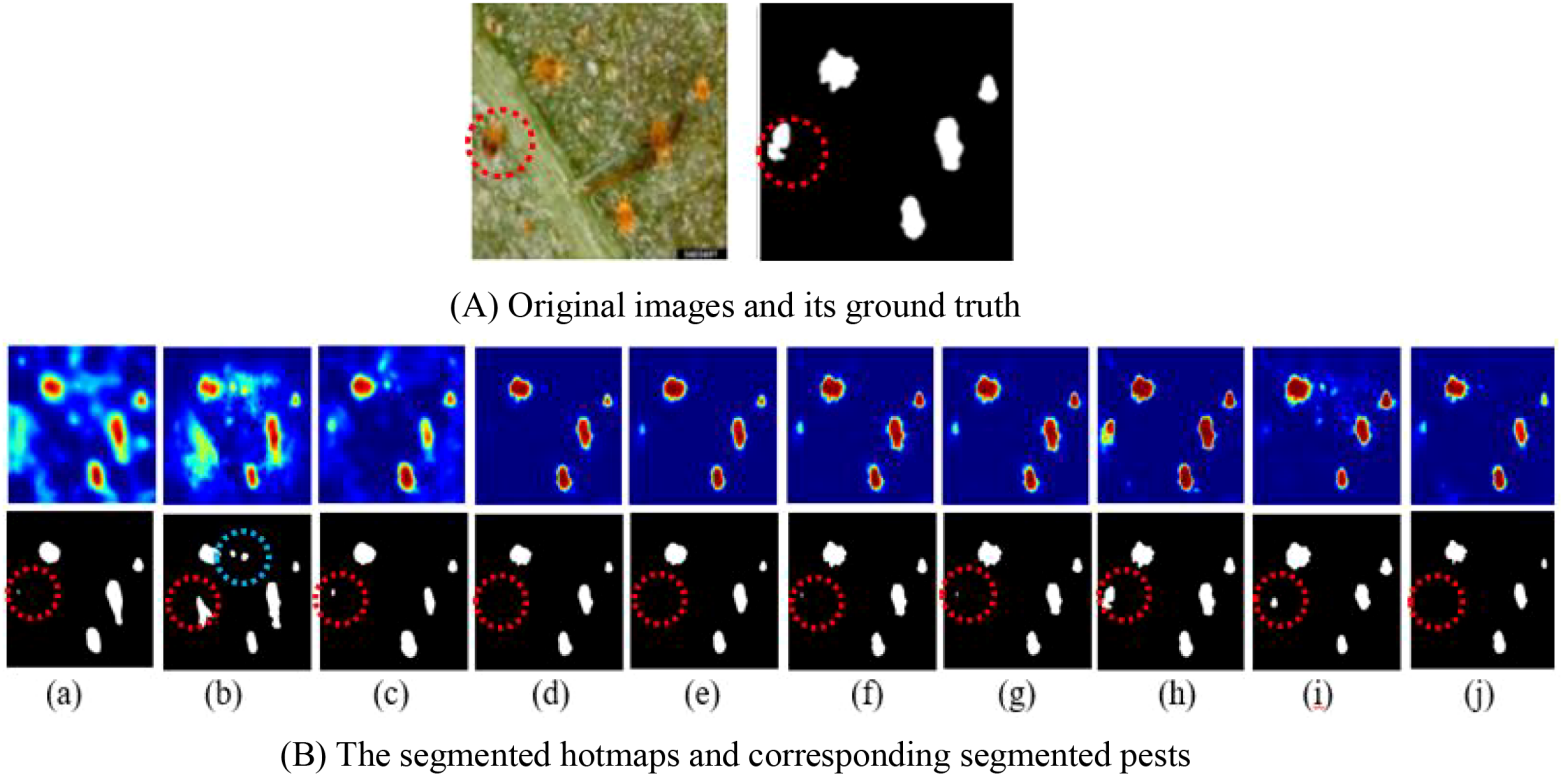
Segmented pest comparison for MSAF-VMUNET variants: **(A)** Original image and ground truth; **(B)** Segmented heatmaps (top) and pest results (bottom, (a-j)). Red circles: detailed comparison regions; blue circles: false comparison regions.

Different from VM-UNet, MSAF-VMUNet has five improvements, i.e., MSVSS, IAF, AVSS, IPM and IPE. To intuitively demonstrate their impact on PPIS performance, some visual experiments are conducted by the variant models of MSAF-VMUNet under the above same experimental conditions. The segmented pest hotmaps and corresponding pests by 10 variants of MSAF-VMUNet are shown in **Figure 6B**, where (a) U-Net with IPM and IPE, (b) VM-UNet with max-pooling and upsampling, (c) With VSS and with cross-entropy loss function ( σ=1), (d) Without IAF and AVSS, (e) Without CSA and with VSS, (f) Without IAF, (g) Without AVSS, (h) MSVSS is replaced by VSS, (i) IPM is replaced by max-pooling, (j) IPE is replaced by up-sampling.

The ablation experiments are conducted to verify the contribution of each proposed component. The model variants are constructed in accordance with the logical design process: starting from the baseline VM-UNet architecture, the core innovations of the model are gradually integrated. First, MSVSS is used to enhance feature extraction, then IAF is used to improve feature propagation and patch operations (IPM and IPE), and the global feature optimization is integrated through AVSS bottleneck. This step-by-step approach allows for the isolation of the influence of each component. The results are summarized in **Table 4**.

**Table 4:** The different variants and corresponding segmented results.

Model variant	Encoder/decoder	Skip connection	Bottleneck	Precision (%)	Recall (%)	mIOU (%)
1. Baseline	VSS	Concatenation	VSS	75.78	73.28	64.13
2. +MSVSS	MSVSS	Concatenation	VSS	77.25	74.53	65.28
3. +IAF	MSVSS	IAF Modules	VSS	78.40	73.73	65.41
4.+IPM & IPE	MSVSS	IAF Modules	VSS	78.46	73.77	65.45
5. MSAF-VMUNet	MSVSS	IAF Modules	AVSS	79.17	73.42	65.58

The results in **Table 4** reveals a consistent performance improvement with each integrated component, where MSVSS contributes the most substantial precision gain (+1.47%), establishing a strong multi-scale feature extraction foundation, the subsequent IAF module further elevates precision to 78.40%, which is achieved at the cost of a marginal recall drop, reflecting its selective filtering mechanism. This trade-off is effectively optimized in the final configuration by the AVSS module, which enhances global feature representation. The complete MSAF-VMUNet achieves an optimal balance, attaining 79.17% precision and 65.58% mIoU, and validating the complementary nature and synergistic integration of all proposed components.

### 4.9 Impact of computational complexity

To illustrate that the computational complexity of the proposed model is a linear complexity of O(n), while Transformers the quadratic complexity of O(n²), we compare MSAF-VMUNet against three representative baseline models: U-Net, Trans-FCLIE, and VM-UNet on a Single GPU. **Table 5** presents the comparison results.

**Table 5:** Inference performance comparison on a single GPU.

Model	Precision (%)	mIoU (%)	FPS (PS	Memory (GB, ↓G
U-Net	64.66	52.18	**32.1**	**1.2**
Trans-FCLIE	75.09	64.89	14.8	3.5
VM-UNet	75.78	64.15	26.5	2.1
Ours	**79.17**	**66.58**	27.9	2.3

The bold values provided are the results of the method MSCNN-VSS proposed in the paper.

The results in **Table 5** verify that the Transformer-based model (Trans-FCLIE) is slower (14.8 FPS) due to its quadratic complexity, our method MSAF-VMUNet operates at 27.9 FPS, which is nearly twice as fast, while simultaneously achieving the highest Precision and mIoU. This demonstrates MSAF-VMUNet is an ideal balance of high performance and efficiency for real-world agricultural deployment.

### 4.10 Discussion

Based on the experimental results shown in **Figures 4**-**6** and **Tables 3** and **4**, it can be seen that MSAF-VMUNet demonstrates superior PPIS performance under challenging field conditions, including small pests with blurry and low contrast scenes. Compared with the suboptimal model Trans-FCLIE, MSAF-VMUNet achieves higher segmented results. The reasons are three key factors: (1) It can capture global and remote semantic features, and can effectively extract significant visual features that distinguish small pests from complex backgrounds. (2) The integration mechanism of MSVSS, IAF, AVSS, IPM and IPE specifically addresses the challenge of small pest segmentation and enhances the recognition of sub-pixel-level fine-grained features such as antennas and feet. (3) The channel-spatial attention mechanism combined with advanced feature fusion strategies effectively reduces information loss during the downsampling process of feature maps, thereby improving the PPIS accuracy of multiscale pests.

MSAF-VMUNet with MSVSS block incorporates dilated convolutions to extract multi-scale features and enhance robustness to pest size and shape variations, uses IAF module dynamically filters encoder features before fusion, replacing standard skip connections to strengthen feature representation across diverse pest appearances.

## 5 Conclusion

Taking advantage of the strengths of the Mamba architecture in long sequence modeling and efficient global context extraction through SSM, this study proposes MSAF-VMUNet for field paddy PPIS. This model effectively addresses the key issues in PPIS by integrating MSVSS, IAF, and AVSS modules, which can enhance the segmentation performance of small pests under complex field conditions, while maintaining the linear computational efficiency of Mamba. This model introduces a multi-scale attention fusion mechanism, which can simultaneously focus on different scale context information of pest and disease characteristics and adaptively emphasize key areas, thereby achieving more accurate and robust pixel-level segmentation of pest damage areas in complex rice field images. It effectively overcomes the shortcomings of CNN in long-distance dependency modeling and the shortcomings of Transformer in computational efficiency and local detail capture. Comprehensive experiments conducted on the paddy image set in IP102 dataset verify the effectiveness of MSAF-VMUNet in real-world scenarios, demonstrating its powerful capabilities in handling occlusion, multi-scale pest variations, and various field environments. This model offers a practical, accurate and efficient automatic pest monitoring solution to support sustainable crop management. Future work will advance this research from two key directions. Firstly, inspired by the surface feature detection method (Chen et al., 2025), a cross-modal fusion framework will be developed to align image and text embeddings using contrastive learning. Secondly, knowledge graph technology (Liu et al., 2025) and high-resolution processing technology will be explored to enhance the detection of small pests, and it is possible to conduct large-scale training using supercomputing infrastructure.

## Data Availability

The original contributions presented in the study are included in the article/supplementary material. Further inquiries can be directed to the corresponding author.
